# Feasibility, acceptability and outcomes of blended care with smartphone-based ecological momentary assessment and intervention in schizophrenia spectrum disorders: A pilot single-arm trial

**DOI:** 10.1192/j.eurpsy.2025.53

**Published:** 2025-08-26

**Authors:** K. Böge

**Affiliations:** Charite, Berlin, Germany

## Abstract

**Abstract:**

Delusions are one of the core symptoms of schizophrenia spectrum disorders (SSD). Traditional Cognitive Behavioral Therapy (CBT) approaches are less effective for delusions, require significant resources, and specialized staff training. Symptom-specific therapy approaches, which target factors involved in the development and maintenance of psychotic symptoms, provide a valid alternative. Digital technologies, such as ecological momentary assessment (EMA) and ecological momentary intervention (EMI), are gaining attention in mental health, providing enhanced assessment and intervention opportunities. The present single-arm trial aimed to investigate the feasibility, acceptability, and preliminary outcomes of a smartphone-based blended EMA/I psychological therapy approach - DICE - focusing on improving coping strategies for delusions in SSD. In total, *N* = 10 participants received four face-to-face therapy sessions alongside German university-level treatment-as-usual over an intervention period of four to six weeks. Feasibility was assessed by completion rates of the EMA/I questionnaires, use of the application between sessions and recruitment rates. Acceptability was assessed by a satisfaction questionnaire, open feedback, and analysis of adverse effects. Clinical outcomes included self-rated and rater-based intensity and distress of delusions and comorbid symptoms at pre- and post-intervention. Findings supported the feasibility and acceptability of the DICE intervention, with high retention (10/13 participants; 77%) and completion rates for the EMA- (59%) and EMI-questionnaires (72%), as well as a high protocol adherence (90-97%), exceeding all predefined benchmarks. Open feedback indicated good satisfaction, with all participants using the application between sessions, reflecting a high engagement level. Clinical outcomes displayed relevant changes in ameliorating the intensity of delusions when being measured by the Psychotic Symptom Rating Scales as well as by the Green Paranoid Thought Scale, and self-rated improvements in distress and depressive symptoms. Changes in the intensity and distress of delusions might be explained by improved coping behaviour. Further research with control conditions is needed to validate findings and analyze the efficacy as well as mechanisms of actions of the DICE intervention in a fully powered trial.

**Disclosure of Interest:**

None Declared

